# A Case of Rhizomelic Chondrodysplasia Punctata in Newborn

**DOI:** 10.1155/2014/879679

**Published:** 2014-03-09

**Authors:** Nalan Karabayır, Gonca Keskindemirci, Erdal Adal, Orhan Korkmaz

**Affiliations:** ^1^Pediatrics Department, Bakırköy Maternity and Children Education and Research Hospital, Kartaltepe mah Aksoy sok. Petrol Sitesi 6/11 Bakırköy, Istanbul, Turkey; ^2^Radiology Department, Bakırköy Maternity and Children Education and Research Hospital, Turkey

## Abstract

Rhizomelic chondrodysplasia punctate (RCDP) is a rare autosomal recessive peroxisomal disease. The main features of the disease are shortening of the proximal long bones, punctate calcifications located in the epiphyses of long bones and in soft tissues around joints and vertebral column, vertebral clefting, dysmorphic face, and severe growth retardation, whereas cervical spinal stenosis may also rarely be present. Imaging of the brain and spinal cord in patients with this disorder may aid prognosis and guide management decisions. We report the newborn diagnosed as CDP with cervical stenosis. Our aim is to discuss current knowledge on etiopathogenesis as well as radiological and clinical symptoms of diseases associated with CDP.

## 1. Introduction

Rhizomelic chondrodysplasia punctata (RCDP) is a rare disorder of peroxisomal metabolism, with an estimated incidence 1 : 100.000. There are 3 genetic subtypes. RCDP type 1 (OMIM 215100), caused by mutations in the PEX7 gene, is the most common type. RCDP type 2 (OMIM 222765) and 3 (OMIM 600121) are single enzyme deficiencies in the plasmalogen biosynthesis pathway. RCP type 2 arises secondary to mutations in the acyl-CoA:dihydroxyacetone phosphate acyltransferase (DHAPAT) gene, and RCP type 3 arises from mutations in the alkyl-dihydroxyacetone phosphate synthase (ADAPS) gene [1, 2]. The main features of the disease are shortening of the proximal long bones, punctate calcifications in the metaphysis and epiphysis of long bones and the thoracic and lumbar vertebrae, dysmorphic face, and severe growth retardation. Cervical stenosis is very rarely reported in rhizomelic CDP cases [1, 3]. Because of underlying vertebral abnormalities, spinal stenosis, often seen together with brachytelephalangic chondrodysplasia punctata, can cause progressive neurological findings. Here, we report a case of RCDP with cervical spinal stenosis in newborn.

## 2. Case

**Figure 1:**
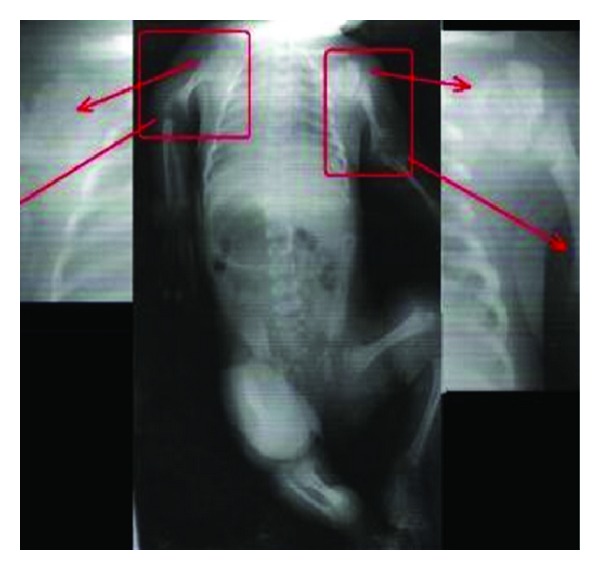
Proximal shortness, thick and short diaphyses, large and irregular metaphyses, and punctate calcifications in the epiphyses in the long bones and coronal clefts not included in the vertebral bodies.

**Figure 2:**
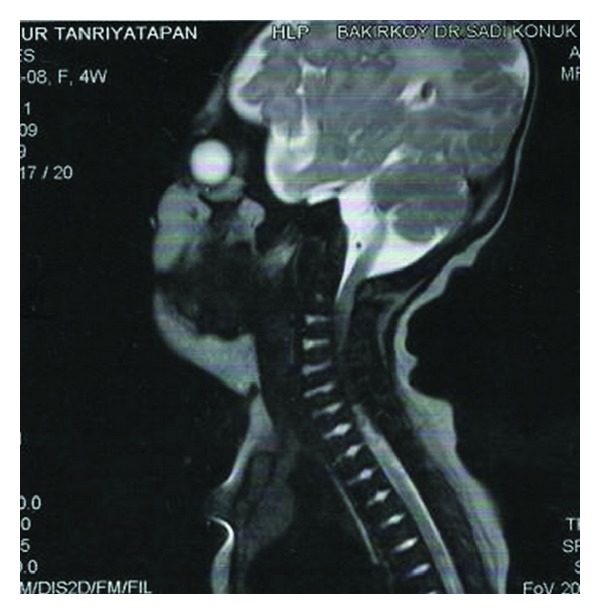
On the cervical spinal MRI, spinal stenosis at C4-5, C5-6, and C6-7 levels.

The term infant was admitted to the neonatology department because of its atypical facial appearance and extremity anomalies at the 2nd hour of her life. The female infant was born at 38 weeks of gestation from the fourth pregnancy of a healthy 22-year-old mother and a 30-year-old related father. The mother was under routine prenatal follow-up during pregnancy. She did not have any chronic disease and there was no history of exposure to any known embryopathic agents and, in particular, no warfarin therapy or alcohol use had been given. Prenatal ultrasonographic assessments reported proximal limb shortening. The mother had two miscarriages, as well as a baby with skeletal abnormalities who was aborted at 22 gestational weeks and a healthy male child. On the physical examination, BW was 3200 gm (50–75 percentiles), height was 50 cm (75–90 percentiles), head circumference was 35 cm (75–90 percentiles), the neck was deviated to the right, and there was a depressed nasal bridge and a highly arched palate. There was shortness of the upper extremities and flexion contractures in all extremities. The infant's systemic examination was otherwise normal. In the skeletal survey performed, there were proximal shortness, thick and short diaphyses, and large and irregular metaphyses in the long bones and normal fingers. The radiological findings of the patient were compatible with CDP with punctate calcifications in the epiphyses and coronal clefts in the vertebral bodies (Figure 1). Complete blood count, biochemical parameters, and abdominal ultrasonography were all normal. Secundum ASD and thin PDA were detected on echocardiography. Cavum vergae and minimal dilatation in the right ventricle were observed on cranial ultrasonography. On the cranial MR investigation, both lateral ventricles and the third ventricle were found to be larger than normal and there was atrophy in the left temporal lobe. On the cervical spinal MRI, spinal stenosis was detected at C4-5, C5-6, and C6-7 levels (Figure 2). Bilateral nuclear cataract was seen on the ophthalmological examination. Her karyotype test was normal (46, XX). Blood amino acid and urinary organic acid levels were unremarkable, and anti-DNA and ANA tests of her mother revealed negative results. Further biochemical studies showed high phytanic acid (9.2; *n* < 5.28 *μ*mol/L) and low plasmalogen (3.2; *n* > 6.6) levels. RCDP1 was diagnosed based on clinical, biochemical, and radiological criteria. Parents were informed about this disease, and genetic counseling was given. The case is now 2 months of age, weighing 4800 gr, and is fed orally. She was operated at the age of 1 month for bilateral cataract. She has been in follow-up for spinal stenosis and probable nutritional problems. Echocardiography was planned to monitor the size of heart defect after 6 months.

## 3. Discussion

Chondrodysplasia punctata (CDP) is a rarely occurring skeletal dysplasia characterized by stippled, punctuate calcifications around joints and within cartilages [1]. CDP is associated with a number of disorders, including inborn errors of metabolism, involving peroxisomal and cholesterol pathways, embryopathy, and chromosomal abnormalities [2–7]. Several classification systems of the different types of CDP have been suggested earlier. More recently, the biochemical and molecular basis of a number of CDP syndromes has recently been elucidated and a new aetiological classification has emerged [2]. Rhizomelic chondrodysplasia punctata is a disorder caused by abnormal peroxisomal function which can be mediated both through disorders of biosynthesis, for example, peroxisomal assembly (RDCP1), and by single enzyme defects, affecting plasmalogen synthesis (RCDP2, RCDP3). Clinically, RCDP1, RCDP2, and RCDP3 are characterized by rhizomelic shortening, mainly affecting the humerus, facial dysmorphism, seizures, cataracts, and joint contractures. Growth and development are severely restricted. Life expectancy is considerably reduced [1, 2, 8]. Pathognomonic finding for RCDP is a reduced level of plasmalogens with normal very long chain fatty acids (VLCFA) [1]. The following situations should be considered in the differential diagnosis of CDP: peroxisomal diseases (Zellweger Syndrome, adrenoleukodystrophy, and infantile Refsum disease), maternal disease, and exposure to warfarin, Smith-Lemli-Opitz Syndrome, and foetal alcohol syndrome [2, 9, 10]. There was no history of maternal drug or alcohol use and no symptoms or positive laboratory test that indicated autoimmunological disease in the mother. Because our case had high phytanic acid level and autosomal recessive inheritance, she was diagnosed as RCDP1.

Spinal stenosis is a frequent sign of bone dysplasia, while it is rarely reported in rhizomelic CDP cases [11–13]. Cervical spinal stenosis, which in some cases leads to cord compression and myelopathy, has been described in chondrodysplasia punctate of rhizomelic, brachytelephalangic, and Conradi-Hunermann types [13]. Because patients with RCDP often demonstrate upper and lower extremity spasticity in the absence of spinal cord involvement, diagnosis of cervical spinal stenosis secondary to RCDP may be difficult. In addition, it is sometimes hard to establish the clinical finding of spasticity because multiple joints have limited range of motion [14]. Also, a recent study found an association between overall disease severity and the presence of abnormalities on brain magnetic resonance imaging [15]. Because neuroimaging can provide prognostic information, it is important to look for cervical stenosis in these patients. These patients may be screened with conventional radiographs of the cervical spine and cases with radiographs suggestive of spinal stenosis and physical examinations compatible with myelopathy may be further evaluated with MRI when clinically indicated [14]. In our case, cervical stenosis was detected on the spinal MRI investigations performed for this purpose. But our patient who had cervical stenosis still has no neurological findings.

Spasticity, psychomotor retardation, growth retardation, seizures, thermoregulatory instability, feeding difficulty, recurrent otitis media, and pneumonia have been reported in CDP cases [11]. Our case was still fed orally in spite of the feeding difficulty, and her weight was 4800 gm (50–75 p) at two months. So, our patient was described as mildly affected.

Prenatal diagnosis of RCDP is possible from the first trimester onwards by demonstration of peroxisomal dysfunction in cultured chorionic villous or amniotic fluid cells [3]. Genetic counseling was given to parents of our case.

In conclusion, cervical spinal stenosis is a rare anomaly in rhizomelic chondrodysplasia punctata cases, which may cause neurological findings. Imaging of the brain and spinal cord in patients with this disorder may aid prognosis and guide management decisions.
